# Evaluation of antimicrobial activity of *Malus domestica* fruit extract from Kashan area

**Published:** 2013

**Authors:** Sara Jelodarian, Abdolrasoul Haghir Ebrahimabadi, Fereshteh Jookar Kashi

**Affiliations:** 1 *Department of Hortiscience, Faculty of Agriculture and Natural Sources, Science and Research Branch, Islamic Azad University, Tehran, I. R. Iran*; 2 *Essential Oils Research Institute, University of Kashan, Kashan, I. R. Iran*

**Keywords:** Apple, Antimicrobial, Extract, *Malus domestica*

## Abstract

**Objective: **Many species of plants present noticeable potency against human bacterial and fungal pathogens.

**Materials and Methods: **In the current study, antimicrobial activity of the fresh fruits of 4 cultivars (A to D) of *Malus domestica *cultivated in the Qamsar area of Kashan, Iran was investigated. The disk diffusion and minimal inhibitory concentration (MIC) evaluation antimicrobial activity tests were performed.

**Results: **The samples showed moderate antimicrobial activities with inhibition zones from 11 to 16 mm in these tests. Cultivar D with inhibition zones of 16, 14, and 12 mm for *E. coli*, *S. epidermidis* and *K.* *pneumoniae*, respectively exhibited the best results in these tests. Cultivar A also showed a zone of inhibition of 11 mm against *P.* *aerouginosa*.

**Conclusion: **Moderate antimicrobial activities were observed for the studied apple cultivars.

## Introduction

Fruits and vegetables are the main food source for some essential nutrients and also include a series of bioactive components, which might have multiple effects in the fields of health (Yigit et al., 2009 ▶). Among fruits, apples are consumed in different forms, i.e., fresh, in juices, and cider.

Apples have several useful aspects for everybody’s health in regard to their high capacity in phenolic contents (Alberto, 2006 ▶). Phenolic compounds include a significant class of phytochemicals that possess various biological functions such as astringent, antioxidant, anticancer, anti-inflammation, and antibacterial activity (Jeong et al., 2009 ▶; Rubnov et al., 2001 ▶; Vaya & Mahmood, 2006 ▶; Ryu et al., 1998 ▶).

In addition, incidence or survival/growth of *Listeria monocytogenes*, *Listeria innocua*, *Salmonella serovars*, and *Escherichia coli* O157:H7 in fruit juices and apple cider has been demonstrated (Raybaudi-Massllia et al., 2009 ▶; Ceylan et al., 2004 ▶; Harris et al., 2003 ▶; Ingham et al., 2006 ▶; Miller & Kaspar, 1994 ▶; Raybaudi-Massilia et al., 2006 ▶).

*Malus domestica* Borkh is a kind of fruit that is consumed all over the world (Shoji et al., 2004 ▶). Our samples are ancient with medium size and circular shape. The yellow–pink skins are thin, rather wax-like, and their white ﬂesh is soft, juicy, aromatic, and sweet. Because of staying on the tree, the skin color of these 4 apple cultivars changes gradually and becomes red.

The objective of this study was to measure the antimicrobial potential of these fruit extracts.

## Materials and Methods

**Fruit collection**

Fresh fruit samples from Hossain, Sayyed Babaeei, Shekareh, and Golab apple cultivars were collected in the Qamsar area of Kashan, Iran, in the June 2008 when the fruit had just been harvested. They were randomly named as A, B, C and D.

**Extraction procedure**

Apples were characterized by a plant taxonomist, immediately transported to the laboratory, washed, dried, cut manually with a knife into small pieces, whole fruit except seeds extracts were obtained using a kitchen-type blender (Moulinex, France), and concentrated with a rotary evaporator.

**Antimicrobial activity**

*Microbial strains*

The extracts of *Malus domestica* were individually tested against a panel of eleven microorganisms. Following microbial strains were provided by Iranian Research Organization for Science and Technology (IROST) and were used in this research: *Pseudomonas aeruginosa* (ATCC 27853), *Bacillus subtilis* (ATCC 6633), *Escherichia coli* (ATCC 10536), *Staphylococcus aureus* (ATCC 29737), *Klebsiella pneumonia* (ATCC 10031), *Staphylococcus epidermidis* (ATCC 12228), *Shigella dysenteriae* (Persian Type Culture Collection or PTCC 1188), *Proteus vulgaris* (PTCC 1182), *Salmonella paratyphi-A serotype* (ATCC 5702), *Candida albicans* (ATCC 10231), and *Aspergillus niger* (ATCC 16404).

Bacterial strains were cultured overnight at 37 °C in nutrient agar (NA) and fungi were cultured overnight at 30 °C in sabouraud dextrose agar (SDA).

Bacterial strains were cultured overnight at 37 °C in nutrient agar (NA) and *Candida albicans* and *Aspergillus niger* were cultured overnight at 30 °C in sabouraud dextrose agar (SDA).

*Disc diffusion assay*

Determination of antimicrobial activity of extracts was accomplished by agar disc diffusion method (NCCLS, 1997 ▶).The extracts were dissolved in dimethyl sulfoxide (DMSO) to a ﬁnal concentration of 30 mg/ml and ﬁltered by 0.45 µm Millipore ﬁlters for sterilization.

Antimicrobial tests were carried out by the disc diffusion method reported by Murray et al., (1999) using 100 µl of suspension containing 10^8 ^CFU/ml of bacteria, 10^6 ^CFU/ml of yeast and 10^4^ spore/ml of fungi spread on the nutrient agar (NA), sabouraud dextrose (SD) agar, and potato dextrose (PD) agar mediums, respectively.

The discs (6 mm in diameter) were impregnated with 10 µl of the essential oil or the extract solution (300 µg/disc) and DMSO (as negative control) were placed on the inoculated agar.

The inoculated plates were incubated for 24 h at 37 °C for bacterial strains and 48 h and 72 h at 30 °C for yeast and mold isolates, respectively. Gentamicin (10 µg/disc) and rifampin (5 µg/disc) were used as positive controls for bacteria and nystatin (100 IU) for fungi.

The diameters of inhibition zones were used as a measure of antimicrobial activity and each assay was repeated twice.

*Micro-well dilution assay*

Bacterial strains and yeast sensitive to the extracts in disc diffusion assay were studied for their minimal inhibition concentration (MIC) values using micro-well dilution assay method (Güllüce et al., 2004 ▶).

The inocula of the bacterial strains and fungi were prepared from 12 h and 18 h broth cultures, respectively and suspensions were adjusted to 0.5 McFarland standard turbidity. The extracts of apples dissolved in 10% DMSO solution were ﬁrst diluted to the highest concentration (5 mg/ml) to be tested, and then serial two-fold dilutions were made in a concentration range from 0.078 to 5 mg/ml in 10 ml sterile test tubes containing brain heart infusion (BHI) broth for bacterial strains and sabouraud dextrose (SD) broth for yeast. The 96-well plates were prepared by dispensing 95 µl of the cultures media and 5 µl of the inoculum into each well.

A 100 µl aliquot from the stock solutions of the extracts was initially prepared at the concentration of 5 mg/ml and added into the ﬁrst wells. Then, 100 µl volumes from their serial dilutions were transferred into six consecutive wells. The last well containing 195 µl of the cultures media without the test materials and 5 µl of the inoculum on each strip was used as the negative control. The ﬁnal volume in each well was 200 µl. 

Gentamicin and rifampin for bacteria and nystatin for yeast were used as standard drugs for positive control in conditions identical to tests materials. The plates were covered with sterile plate sealers. Contents of each well were mixed on plate shaker at 300 rpm for 20 s and then incubated at appropriate temperatures for 24 h. Microbial growth was determined by the presence of a white pellet on the well bottom and conﬁrmed by plating 5 µl samples from clear wells on NA medium. The MIC value was deﬁned as the lowest concentration of the extracts required for inhibiting the growth of microorganisms. All tests were repeated two times.

## Results

The antimicrobial activity of extract of *Malus domestica* was evaluated against a set of eleven microorganisms and their potencies were assessed qualitatively and quantitatively by the presence or absence of inhibition zones, the corresponding zone diameters, and MIC values. The results given in Table 1 indicate that at the tested concentrations, the extract of cultivars D and A have notable antimicrobial activity against tested microorganisms. *P. aerouginosa* was sensitive to cultivar A and inhibition zone of 11 mm was reported.

*E. coli*, *S. epidermidis*, and *K. pneumonia* sets were sensitive to cultivar D and Inhibition zone of 16, 14, and 12 mm was reported. However, cultivars B and C did not show any activity against the microorganisms tested.

**Table 1:** Antimicrobial activities of extracts of four apple cultivars against tested microbial

	**Extracts**								**Antibiotics**					
	**A**		**B**		**C**		**D**		**Rifampin**		**Gentamicin**		**Nystatin**	
**Test microorganisms** **Gram-negative bacteria, Gram-positive bacteria and fungi**	DD	MIC	DD	MIC	DD	MIC	DD	MIC	DD	MIC	DD	MIC	DD	MIC
***P. aeruginosa***	11	7.81	-	-	-	-	-		-	-	8	500	NA	NA
***E. coli***	-	-	-	-	-	-	16	500	11	125	20	500	NA	NA
***K. pneumoniae***	-	-	-	-	-	-	12	500	7	500	22	250	NA	NA
***P. vulgaris***	-	-	-	-	-	-	-	-	10	250	20	500	NA	NA
***S. paratyphi-A serotype***	-	-		-	-	-	-	-	-	-	20	250	NA	NA
***B. subtilis***	-	-	-	-	-	-	-	-	13	250	18	7.81	NA	NA
***S. aureus***	-	-	-	-	-	-	-	-	10	500	24	500	NA	NA
***S. epidermidis***	-	-	-	-	-	-	14	125	40	125	39	500	NA	NA
***S. dysenteriae***	-	-	-	-	-	-	-	-	-	-	25	500	NA	NA
***C. albicans***	-	-	-	-	-	-	-	-	NA	NA	NA	NA	33	125
***A. niger***	-	-	-	-	-	-	-	-	NA	NA	NA	NA	27	31.2

^a ^DD (Disc diffusion method), Inhibition zones in diameter (mm) around the impregnated discs.

^b ^MIC (Minimal Inhibition concentrations as μg/ml).

NA (Not applicable).

## Discussion

In general, the Gram-positive strains of bacteria tested appeared to be more sensitive to the extracts. However, this study also records a significant susceptibility of some of the examined Gram-negative bacteria. According to literature, the antimicrobial activity could be influenced by the phenolic compounds (Alberto et al, 2001 ▶; Alberto et al, 2002 ▶; Alberto et al, 2004 ▶). Moreover, Alberto (2006) ▶ reported that there is a direct relationship between phenolic content and antibacterial effect in four apple cultivars (Xiangyang et el., 2003 ▶) and their polyphenol extracts had stronger inhibition effects on the bacteria. An *in vivo* assay is necessary to confirm the antimicrobial activities of *Malus domestica*, which could be usefully applied to the food, pharmaceuticals, and cosmetics industries. Isolation of the gene responsible for the antimicrobial activity would be an interesting future study topic aimed at identifying the molecule generating the desirable efficacy. As a whole, these samples had moderate antimicrobial activities against a set of eleven microorganisms. 

Many plant species are currently used as sources of nutritional additives because of their antioxidant and antimicrobial properties that increase immunity to some diseases. This work, as the ﬁrst study on the *in vitro* antimicrobial activity of four cultivars, reports appreciable antimicrobial potential for these samples. These ﬁndings candidate them as a good cases for more in-depth studies. Moreover, these cultivars can be proposed in food industries, as flavor and preservative or in cosmetic-health industries as antimicrobial agents. We wish our future research lead to the identiﬁcation and structure elucidation of biologically active molecules present in their extracts.

## Conﬂict of interest

The authors declare that there are no conﬂicts of interest.
